# Evaluation of *Ganoderma lucidum* Across Varieties and Growth Stages: Integrating Chromatographic Profiling, Bioactivity Correlation, and In Silico Simulations

**DOI:** 10.3390/foods15122071

**Published:** 2026-06-08

**Authors:** Xianxian Miao, Shuai Zhou, Jinyan Wang, Jie Feng, Zhenhao Li, Guoliang Zhang, Na Feng, Jingsong Zhang

**Affiliations:** 1 Institute of Edible Fungi, Shanghai Academy of Agricultural Sciences, Shanghai 201403, China; rongxuan_miao@126.com (X.M.); zhoushuai@saas.sh.cn (S.Z.); wangjinyan@saas.sh.cn (J.W.); fengjie@saas.sh.cn (J.F.); 2 Zhejiang ShouXianGu Botanical Drug Institute, Hangzhou 321200, China; zhenhao6@126.com (Z.L.);

**Keywords:** *Ganoderma lucidum*, quality evaluation, 5*α*-reductase inhibitory activity, molecular dynamics simulations, systematically quantitative fingerprint method, chemometrics

## Abstract

To address the lack of a comprehensive quality control system for *Ganoderma lucidum*, we developed an integrated evaluation strategy across four varieties and three growth stages. This system integrates the targeted screening of anti-benign prostatic hyperplasia (BPH) triterpenoids acting on 5*α*-reductase type 2 (SRD5A2) with a chemical consistency assessment utilizing systematic quantitative fingerprint method (SQFM) and chemometrics. UPLC-Q-TOF-MS/MS identified 85 triterpenoids in the samples. An orthogonal partial least squares (OPLS) regression was utilized to screen seven chromatographic peaks that positively correlated with SRD5A2 inhibitory activity. Three principal bioactives were structurally identified as ganoderic acids DM and B, and ganoderenic acid A, which demonstrated significant in vitro SRD5A2 inhibition rates of 61.16 ± 1.87%, 36.41 ± 1.10%, and 41.82 ± 2.09%, respectively. Molecular dynamics simulations and averaged weak interaction analysis revealed that these compounds exert potent enzyme inhibition via hydrogen bonds and hydrophobic interactions with distinct SRD5A2 amino acid residues. The SQFM-chemometrics quality system confirmed ten samples reached Grade 6 or above, identifying the H3 variety at the initial stage as possessing the highest active ingredient content and optimal overall quality. This integrated framework enables rapid bioactive discovery and robust standardization for *G. lucidum*-based functional foods, thereby facilitating their industrial development.

## 1. Introduction

As a traditional edible and medicinal fungus in China [1], *Ganoderma lucidum* is rich in diverse bioactive compounds such as triterpenoids [2] and polysaccharides [3], exhibiting significant pharmacological properties including anti-androgenic [4], anti-tumor [5,6], and anti-inflammatory [7] effects. In recent years, driven by the growing demand in the international market, artificial cultivation has become the primary approach for acquiring this resource [8]. However, dynamic metabolic fluctuations across different growth stages cause major variations in the quality and bioactivity of *G. lucidum* products [4]. Given that its pharmacological efficacies rely heavily on these dynamic bioactive ingredients, particularly triterpenoids as characteristic biomarkers, the lack of a robust and comprehensive quality control system has emerged as a critical bottleneck hindering the industrialization of *G. lucidum*.

To address this critical issue, constructing an integrated evaluation framework that integrates biological efficacy with chemical profiling represents is a crucial strategy for the holistic quality management of *G. lucidum*. However, the application of this system is constrained by methodological challenges in both biological and chemical evaluations. Biologically, there are a lack of efficient approaches for discovering bioactive components. Traditional phytochemical isolation and bioassay-guided fractionation suffer from notable drawbacks, including low target specificity, a high risk of losing trace active components, and the frequent decrease in or complete loss of bioactivity in isolated compounds [9,10]. Chemically, routine analyses relying on conventional chromatographic or spectroscopic methods are insufficient for rigorous quality control, as the absence of quantitative characterization prevents the reliable screening of high-quality *G. lucidum* samples [11,12].

To overcome these limitations, this study proposes an advanced multidisciplinary approach. For the bioactivity assessment dimension, an anti-benign prostatic hyperplasia (BPH) model was selected. Given that 5*α*-reductase type 2 (SRD5A2) is a crucial enzyme driving BPH pathogenesis [13,14], discovering safe and potent natural bioactive constituents from *G. lucidum* targeting SRD5A2 represents a major research hotspot, supported by the known in vitro inhibitory activities of several triterpenoids, including ganoderic acids TR and DM [4,15]. To quickly screen these inhibitors, correlating the chromatographic fingerprints with bioactivity analysis has emerged as an effective method. By coupling chromatographic fingerprinting with chemometrics, this technique quantifies the correlation between chemical profiles and bioactivities [16,17,18]. Furthermore, when coupled with molecular dynamics (MD) simulations and averaged noncovalent interaction (aNCI) analysis, this approach not only accelerates the screening process but also precisely elucidates target binding modes and underlying molecular mechanisms. For chemical profiling, the systematic quantitative fingerprint method (SQFM) was employed to address the quantitative limitations of standard fingerprinting [19,20]. By utilizing the quantitative similarities (*S*_m_ and *P*_m_) and variation coefficient (*α*) alongside principal component analysis (PCA) and hierarchical cluster analysis (HCA), this method enables qualitative and quantitative assessments to evaluate the chemical consistency of *G. lucidum*.

Building upon this theoretical framework, this study systematically identified the triterpenoids of four *G. lucidum* varieties at three growth stages using ultra-performance liquid chromatography–quadrupole time-of-flight tandem mass spectrometry (UPLC-Q-TOF-MS/MS). A combined quality evaluation approach was established to evaluate the *G. lucidum* samples. This strategy integrates OPLS- and MD-driven targeted screening of anti-BPH active components with a systematic chemical consistency assessment based on SQFM and chemometrics. Ultimately, this combinatorial system mitigates current quality control challenges, offering a methodological tool to support the consistent formulation of *G. lucidum*-based functional foods.

## 2. Materials and Methods

### 2.1. Reagents

Reagents including LC-MS grade formic acid and acetonitrile were sourced from Thermo Fisher Scientific Inc. (Waltham, MA, USA)., whereas chromatographic-grade methanol (MeOH) and acetonitrile, together with analytical-grade anhydrous ethanol and MgCl_2_, were supplied by Sinopharm Chemical Reagent Co., Ltd. (Shanghai, China). Reference standards including ganoderic acids I, C2, C6, G, N, B, AM1, K, A, H, D2, C1, F, J, DM, and TR, lucidenic acids C, N, E, B, A, and D, ganoderenic acids B, H, A, and D, dehydro-lucidenic acid A, deacetyl-ganoderic acid F, danzhi acid E, and ganodermanontriol for UPLC were provided by Shouxiangu Institute of Botanical Medicine. Testosterone (T) and rat liver microsomes were purchased from Anpecloud Experimental Supplies (Shanghai, China) Co., Ltd., and Gaojiaoyan (Beijing, China) Technology Co., Ltd., respectively. NADPH was obtained from Beyotime Biotechnology (Shanghai, China) Co., Ltd.

### 2.2. G. lucidum Materials

#### 2.2.1. Varieties and Liquid Culture

Varieties of *G. lucidum* H1–H4 (Hunong Nos. 1–4) were procured from Institute of Edible Fungi at the Shanghai Academy of Agricultural Sciences. The mycelia were inoculated into a liquid broth containing glucose (30 g/L), yeast extract (3 g/L), KH_2_PO_4_ (2 g/L), and MgSO_4_·7H_2_O (2 g/L). Cultivation was performed in an orbital incubator maintained at 25 °C with continuous agitation at 130 rpm over a 7 days period [21].

#### 2.2.2. Cultivation and Sampling

The substrate of cultivation was composed of 79% wood chips, 20% wheat bran, and 1% gypsum powder by dry weight, which was then mixed with water to reach a moisture content of 60%. The prepared substrate was filled into polypropylene bags (9 cm × 15 cm) by about 1000 g per bag. To ensure complete sterility, the substrate bags underwent moist-heat sterilization in an autoclave (121 °C, 120 min) and were subsequently cooled to room temperature. After inoculation with 20 mL of liquid inoculum into individual cultivation bags, mycelial growth was maintained in a controlled incubator at 22–25 °C with a relative humidity of 60–70% for approximately 30 days. Subsequent fruiting body development was stimulated by maintaining thermal conditions at 30–35 °C, coupled with constant ventilation and water spraying to ensure humidity levels remained at 80–95%. Sampling was performed at three distinct growth stages (S1–S3) over 2–5 months post-inoculation (Figure S1): S1 (initial stage of fruiting body formation, occurring 2.5 months post-inoculation, typified by pileus development at the stipe apex), S2 (mature fruiting body stage, observed at 3.5 months, characterized by pore formation and complete pileus expansion), and S3 (spore-releasing stage, observed at 4.5 months, marked by massive spore release). For each stage, fruiting bodies were collected from five randomly selected bags per group. Finally, the collected samples were dried with hot air at 60 °C and ground using a grinder for subsequent extraction [22].

### 2.3. Sample Extraction

For triterpenoid extraction, exactly 1 g aliquots of the samples were suspended in absolute ethanol (1:20, *w*/*v* ratio) and sonicated for 1 h, thereby facilitating the maximal release of triterpenoids. Subsequent clarification via centrifugation (9000 rpm, 20 min) enabled for the collection of the supernatant. All extractions were prepared in triplicate and individually utilized for HPLC/UPLC-based triterpenoid analysis and bioactivity assays [23].

### 2.4. UPLC-Q-TOF-MS/MS for Chemical Composition Identification and Quantitative Analysis Conditions

#### 2.4.1. Sample Preparation for UPLC Analysis

Solvent evaporation was achieved by exposing a specific volume of the supernatant to a gentle nitrogen stream until completely dry. Reconstitution of the resulting residue was performed with 1 mL of MeOH, followed by vortexing and centrifugation (4000 rpm, 10 min). To secure an exact final concentration of 0.2 mg/mL, the extract was appropriately diluted using MeOH. To further ensure the removal of undissolved matter, the sample was centrifuged (13,000 rpm, 10 min) again, yielding a highly clarified supernatant, 800 µL of which was transferred for UPLC analysis.

#### 2.4.2. Mixed Standard Solution Preparation

Following the reagent details in Section 2.1, independent reference standard solutions were dissolved in MeOH (0.5 mg/mL). A mixed standard solution was then prepared by aliquoting ganoderic acid I (0.3 mL), ganoderic acid C1 (0.15 mL), and the other respective standards (0.2 mL each) into a 25-mL flask, followed by dilution to the mark with MeOH. From this core mixture, a six-point calibration gradient was derived; specified volumes ranging from 10 μL to 10 mL (specifically 10, 40, 100, 400 μL, 2, and 10 mL) were independently diluted to a fixed working capacity of 10 mL. The calibration plots were mathematically derived by correlating the measured peak responses with the known concentrations of each target analyte. The linear regression data, detection limit (LOD) and quantification limit (LOQ) data of the studied compounds are detailed in Table S1.

#### 2.4.3. Chromatographic Conditions

Analyte resolution was executed utilizing a Waters HSS T3 column (100 mm × 2.1 mm, 1.7 µm) maintained at 25 °C, with a constant flow rate of 0.45 mL/min. The binary solvent system comprised aqueous formic acid (0.1%, *v*/*v*) as eluent A, and absolute acetonitrile as eluent B. The specific multi-step linear gradient profile was programmed as follows (time, %B): 0, 20%; 0–2, 20–26.5%; 2–9, 26.5% (isocratic hold); 9–19, 26.5–35%; 19–28, 35–60%; 28–32, 60–70%; 32–37, 70–90%; 37–40, 90–100%; and 40–45, 100% (column flush).

#### 2.4.4. Mass Spectrometric Conditions

For accurate mass determination, a SYNAPT XS quadrupole time-of-flight (Q-TOF) analyzer, interfaced with a negative-mode electrospray ionization (ESI) source, was employed to generate HRMS data. The electrostatic configurations included a capillary voltage of 3 kV and a cone voltage of 35 V, coupled with a ramped collision energy between 20 and 50 eV. Regarding the thermal and gas conditions, the operational environment was controlled with a 150 °C source temperature, a 500 °C desolvation temperature (gas flow: 1000 L/h), and the nebulizer pressure was regulated at 6.0 bar. Full-scan spectral profiles spanning an *m*/*z* range of 50 to 1200.

### 2.5. Chromatographic Conditions for Fingerprint Analysis

The acquisition method and detection conditions for the chemical fingerprinting were conducted according to a previously described protocol [23].

### 2.6. SRD5A2 Inhibitory Activity

#### 2.6.1. Evaluation of SRD5A2 Inhibitory Capacity

To determine the SRD5A2 inhibitory potential of the samples, an existing procedure detailed in the literature [24] was adopted and subjected to necessary practical adjustments. Following systematic screening, the optimal enzyme incubation time was established at 40 min. The enzymatic reaction was initiated by introducing specific aliquots of the test analyte (10 μL), testosterone (10 μL), and NADPH (20 μL) into a 160 μL suspension of rat liver microsomes. This reaction mixture was subjected to a 40 min incubation period at 37 °C in a water bath. The final assay concentrations were strictly standardized: 0.5 mg/mL for the crude extracts, and 0.5 mM for both triterpenoids and the finasteride (positive control). MeOH was supplemented to quench the enzymatic activity, followed by a 30 min centrifugation step at 13,200 rpm, and the recovered supernatant was clarified utilizing a 0.22 μm membrane filter. Quantification was performed using the external standard method with a T calibration curve (y = 8395x + 2666.1, R^2^ = 0.9999, linear range: 2.5–320 μM). The degree of SRD5A2 inhibition exhibited by each test sample was quantified by evaluating the variance in residual T levels between the treated assays and the blank controls, following incubation under the same conditions. All in vitro SRD5A2 inhibition assays were performed in independent biological triplicates.

#### 2.6.2. HPLC Chromatographic Conditions

To quantify the SRD5A2 inhibitory effects, chromatographic profiling was executed on an HPLC platform. Analyte separation was achieved using a YMC-Pack ODS-AQ analytical column (250 × 4.6 mm, I.D., 5 μm, 12 nm) maintained at 25 °C. The system operated under an isocratic elution mode, delivering a binary solvent mixture of MeOH and H_2_O (75:25, *v*/*v*) at a constant flow rate of 1.0 mL/min. Sample aliquots of 20 μL were introduced, and UV absorption was monitored at 242 nm.

### 2.7. Inhibitory Mechanism Based on Molecular Dynamic Simulations and Averaged Noncovalent Interaction

#### 2.7.1. Molecular Dynamics (MD) Simulations

Driven by the GROMACS 2024.2 [25], a series of MD simulations was conducted. The RCSB PDB served as the primary source for the SRD5A2 crystal structure (PDB ID: 7BW1), whereas the PubChem database provided the 3D geometries for ganoderic acids DM, B, and ganoderenic acid A. System preparation involved parameterizing the 7BW1 with the AMBER14SB force field [26] and embedding the resulting complex within a TIP3P explicit solvent environment [27]. Ligand topologies were generated via the GAFF2 force field [28].

Simulations were conducted at 298.15 K and 1 atm, neutralized to physiological salt concentrations. Atomic clashes within the initial complexes were resolved through energy minimization, which stopped once the maximum force fell below 100.0 kJ·mol^−1^·nm^−1^. Backbone stability was then achieved via a 300 ps restrained dynamics run (1-fs step size). For the final data collection, the systems underwent three independent 100 ns production runs configured with a 2 fs temporal resolution. A uniform 1.0 nm cutoff was applied for both van der Waals forces and Particle Mesh Ewald (PME)-based electrostatic calculations [29], while the Parrinello–Rahman pressure coupling method [30] was applied. Finally, standard GROMACS utilities facilitated the trajectory analysis, coupled with the gmx_MMPBSA tool [31] for calculating binding affinities and conducting energy decomposition at the residue level.

#### 2.7.2. Averaged Noncovalent Interactions

Following the MD simulations, an additional 1 ns run under NVT conditions. During this phase, restraints were applied to fix the position of the ligand. The MD trajectory was recorded at 1 ps, generating 1001 frames. Using this composite trajectory, an averaged molecular independent gradient model (amIGM) [32] analysis was performed using the Multiwfn [32,33] program, and the interaction surfaces were visualized and rendered in VMD.

### 2.8. Principles Underlying the SQFM

The SQFM [20] method employs a mathematical evaluation model centered on macroscopic qualitative analysis, quantitative analysis, and controlling fluctuation. Utilizing numerical calculations, it enables the objective and accurate quality characterization and classification of complex traditional Chinese medicine (TCM) systems. In addition, this analytical method serves as a practical tool for evaluating batch consistency and establishing chemical consistency metrics for both TCM raw materials and final products. Crucially, it overcomes the qualitative-only limitations of conventional chromatographic fingerprinting.

Based on the areas of the peaks (x_i_ and y_i_), the SFP and RFP are structurally defined by the vectors $\vec{x}$ = (x_1_, x_2_, x_3_…x_i_…x_n_) and $\vec{y}$ = (y_1_, y_2_, y_3_…y_i_…y_n_). The macroscopic qualitative similarity (*S*_m_, detailed in Equation (1)) serves as a comprehensive indicator of the underlying chemical architecture and constituent distribution, mathematically derived from two sub-parameters: the qualitative similarity index (*S*_F_) and ratio (*S*′_F_). *S*_F_ represents the cosine of the angle formed by vectors $\vec{x}$ and $\vec{y}$, directly reflecting the degree of proportional similarity among the different components. *S*′_F_ is calculated via the balancing of the responses of all peaks to ensure that the contributions of smaller components are included.

(1) $$S_{m}=\frac{1}{2}(S_{F}+S\prime_{F})=\frac{1}{2}\left(\frac{\sum_{i=1}^{n}x_{i}y_{i}}{\sqrt{\sum_{i=1}^{n}x_{i}^{2}}\sqrt{\sum_{i=1}^{n}y_{i}^{2}}}+\frac{\sum_{i=1}^{n}\frac{x_{i}}{y_{i}}}{\sqrt{n\sum_{i=1}^{n}{\left(\frac{x_{i}}{y_{i}}\right)}^{2}}}\right)$$

To assess the total amount of chemical constituents across the entire chromatogram, the macro quantitative similarity (*P*_m_, Equation (2)) is calculated. This measure acts as an overall score, simply combining the quantitative similarity (*P*) and the projection content similarity (*C*). Parameter *C*, representing the alignment of vector $\vec{x}$ onto $\vec{y}$, reflects the overall content similarity between the test sample and RFP; parameter *P* is used to highlight the quantitative fluctuations among the minor components. It is simply the ratio of the total SFP peak areas to those of the RFP. The coefficient of variation (*α*, Equation (3)) is used to assess differences in peak uniformity between the SFP and RFP. *S*_m_, *P*_m_, and *α* make up the main scores of the SQFM method, and are used together to verify sample chemical consistency.

(2) $$P_{m}=\frac{1}{2}\;(C+P)=\frac{1}{2}\left(\frac{\sum_{i=1}^{n}x_{i}y_{i}}{\sum_{i=1}^{n}y_{i}^{2}}+\frac{\sum_{i=1}^{n}x_{i}}{\sqrt{\sum_{i=1}^{n}y_{i}}}S_{F}\right)\times 100\%,$$

(3) $$\alpha =\left|1-\frac{P}{C}\right|,$$

As a practical analytical approach, the SQFM method enables the sensitive detection of tiny chemical changes, which is crucial for ensuring sample consistency and overall chemical stability. This method employs a scoring system where a grade value closer to 1 indicates superior quality consistency. Generally, the grade of qualified materials should fall within the range of grade 1 to 6; a grade of 7–8 indicates that the quality variation beyond the acceptable limit.

## 3. Results

### 3.1. Chemical Components Identification

UPLC-Q-TOF-MS/MS was applied to profile the chemical constituents of *G. lucidum* samples H1–H4 at three fruiting body growth stages (S1: initial; S2: mature; S3: spore-releasing), with the acquired high-resolution mass spectra in negative electrospray conditions ionization mode processed using UNIFI 1.9 software. To accurately identify the chemical constituents, MS^1^/MS^2^ spectra and retention times were matched with a *Ganoderma* compound database and online databases (e.g., ChemSpider and Reaxys). Comprehensive identification data for all compounds are summarized in Table 1. In total, 93 compounds were identified, predominantly comprising triterpenoids (85 out of 93). The remaining components included meroterpenoids (e.g., Ganomycin J (**6**) and Ganomycin B (**80**)), along with several fatty acids and other metabolites. All identified triterpenoids belong to the highly oxygenated lanostane class. Although they share a standard C30 core derived from squalene, complex oxidation and side-chain cleavage create remarkable structural diversity, allowing them to be subdivided based on side-chain length and specific features [34,35]. Specifically, the identified triterpenoids encompass nine C27, two C29 (**28** and **58**), and 56 C30 triterpenoids, as well as the remaining constituent (>C30) modified C30 skeleton containing 31, 32, 34, and 35 total carbons.

### 3.2. OPLS Regression Analysis

#### 3.2.1. Establishment of HPLC Fingerprints

**Figure 1:**
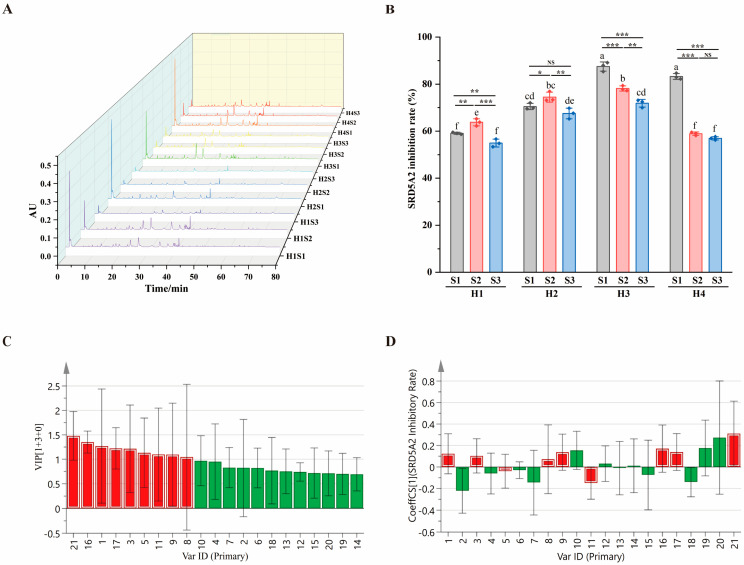
(**A**,**B**): Chromatograms (**A**) and SRD5A2 inhibitory activity (**B**) of crude extracts from H1 to H4 cultivars at three distinct growth stages; (**C**,**D**): VIP (**C**) and coefficient (**D**) plots of OPLS regression. Different letters (a–f) above bars in figure indicate significant differences (*p* < 0.05) by Tukey’s test. * *p* < 0.05, ** *p* < 0.01, *** *p* < 0.001, and NS (no significant difference) between the connected groups.

HPLC analysis of these samples at 252 nm (Figure 1A) established a consistent chemical fingerprint characterized by 21 common peaks (Figure S2). By comparing the retention times with standards (Figure S3), 12 peaks were definitively identified: ganoderic acids C2 (P5), G (P6), B (P8), A (P10), D (P13), F (P19), and DM (P21); ganoderenic acids B (P7), A (P9), D (P12), and F (P20); and lucidenic acid A (P11). These established fingerprints provide a robust data foundation for both the SRD5A2 inhibition correlation analysis and the SQFM-based chemical consistency assessment.

#### 3.2.2. Result of SRD5A2 Inhibitory Activity

As for the in vitro SRD5A2 inhibition, Figure 1B and Table 2 show that all extracts exhibited strong activity, with rates ranging from 54.95% to 87.48%. Notably, the H3S1 sample demonstrated the maximum inhibition (87.48%), whereas H1S3 showed the minimum (54.95%). Comprising the stages revealed distinct bioactivity trends among the varieties: the inhibitory effect of H1 and H2 peaked at stage S2, being significantly higher than at S1 and S3, whereas H3 and H4 displayed the highest activity at stage S1. Taken together, the present study elucidates the correlation between the variations of triterpenoid profiles across three harvesting stages and their specific in vitro SRD5A2 inhibitory potential.

**Table 2:** SQFM evaluation results and SRD5A2 inhibition rates (%).

Fingerprint					SRD5A2 Inhibition Rates (%)
Strain	*S*_m_	*P*_m_%	*α*	Grade	
H1S1	0.987	97.8	0.013	1	59.10 ± 0.37
H1S2	0.981	132.3	0.053	6	63.78 ± 1.23
H1S3	0.939	39.7	0.037	8	54.95 ± 1.35
H2S1	0.877	68.6	0.018	6	70.49 ± 1.10
H2S2	0.858	87.3	0.078	3	74.47 ± 1.73
H2S3	0.807	29.4	0.052	8	67.54 ± 1.84
H3S1	0.932	102.5	0.120	2	87.48 ± 1.59
H3S2	0.933	98.3	0.082	2	78.15 ± 0.90
H3S3	0.918	86.7	0.001	3	71.88 ± 1.35
H4S1	0.978	108.9	0.057	2	83.27 ± 1.02
H4S2	0.970	86.9	0.063	3	58.94 ± 0.57
H4S3	0.954	75.2	0.032	5	56.95 ± 0.56

#### 3.2.3. Correlating Chromatographic Profiles with SRD5A2 Inhibition

An OPLS regression model was built in the SIMCA 14.1 software to explore the relationships between specific chromatographic features and SRD5A2 inhibition. Using the 21 common peak areas as the *X* matrix and the SRD5A2 inhibition rates as the *Y* response yielded a model with excellent fit and predictive ability (R^2^Y = 0.961, Q^2^ = 0.746). A 200-iteration permutation test confirmed the statistical robustness of the established model (Figure S6). Variable Importance in the Projection (VIP) analysis identified nine chromatographic peaks with VIP values > 1 as the main contributors for the differences in SRD5A2 inhibition (Figure 1C). Furthermore, the coefficient plot (Figure 1D) revealed that among these key peaks, seven (P1, P3, P8, P9, P16, P17, P21) exhibited a positive correlation with SRD5A2 inhibitory activity, whereas P5 and P11 displayed a negative correlation. Among the positively correlated peaks, three were structurally identified: P21 (ganoderic acid DM), P9 (ganoderenic acid A), and P8 (ganoderic acid B). Notably, ganoderic acid DM had the strongest effect on the inhibitory activity. To quantify this linear relationship, a regression equation was derived between the inhibition rate (*Y*) and the triterpenoid peak areas (*X_n_*): *Y* = 0.1231*X*_1_ − 0.2213*X*_2_ + 0.1034*X*_3_ − 0.06080*X*_4_ − 0.03944*X*_5_ − 0.0302*X*_6_ + 0.1443*X*_7_ + 0.0723*X*_8_ + 0.1376*X*_9_ + 0.1546*X*_10_ − 0.1501*X*_11_ + 0.0311*X*_12_ − 0.0103*X*_13_ + 0.0115*X*_14_ − 0.0735*X*_15_ + 0.1706*X*_16_ + 0.1396*X*_17_ − 0.1404*X*_18_ + 0.1763*X*_19_ + 0.2741*X*_20_ + 0.3107*X*_21_. Overall, the OPLS regression model linked the fingerprint with SRD5A2 inhibitory activity, finding seven bioactive peaks. Three of these compounds have been identified triterpenoids, while the remaining four unidentified peaks provide targets for future isolation. By overcoming the poor specificity and slow discovery of traditional methods, this approach provides a practical chemometric strategy for the rapid screening of active compounds and targeted efficacy-based quality control of *G. lucidum*.

### 3.3. Bioactivity Evaluation of the Target Active Compounds

The SRD5A2 inhibitory activities of the identified compounds (P5–P13 and P19–P21) were tested in vitro to validate the predictions. As illustrated in Figure S4 and Table S2, seven compounds (P8, P9, P10, P12, P19, P20, and P21) exhibited significant inhibitory effects. Notably, P21 (ganoderic acid DM) demonstrated the most potent activity (61.16 ± 1.87%), even exceeding that of the positive control finasteride (51.74 ± 0.77%). These enzymatic assays align perfectly with the modeling findings, with ganoderic acid DM exhibiting the highest inhibitory potential. Furthermore, P8 and P9 demonstrated moderate inhibitory activity (36.41 ± 1.10% and 41.82 ± 2.09%, respectively), whereas P5–P7, P11, and P13 displayed negligible effects. Overall, these in vitro findings strongly correlate with the OPLS model predictions, substantiating their reliability for the targeted screening of SRD5A2 inhibitors.

### 3.4. Inhibitory Mechanism Based on MD Simulations and Averaged Weak Interactions

#### 3.4.1. MD Simulations

**Figure 2:**
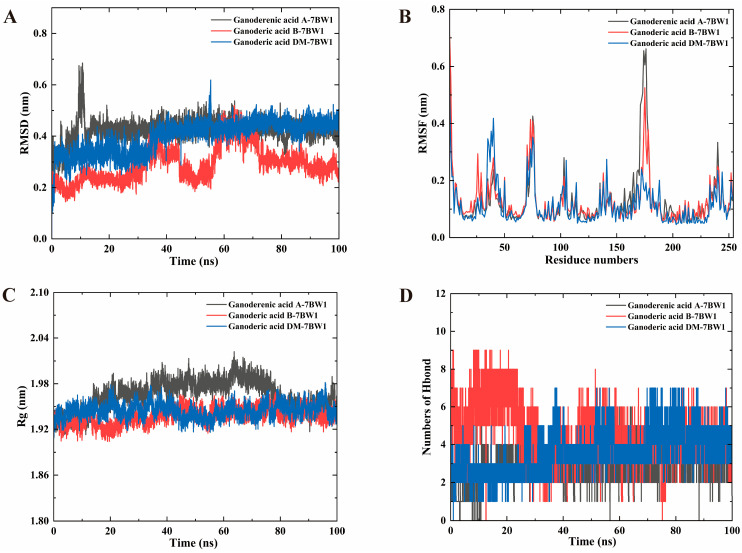
(**A**–**D**): The results of molecular dynamic simulations. RMSD (**A**), RMSF (**B**), Rg (**C**), and Hydrogen bonding (**D**) of complexes.

To investigate the mechanism of SRD5A2 inhibition by triterpenoids, we performed 100 ns MD simulations on these docked complexes. These simulations provided a basis to analyze dynamic binding interactions and confirm the stability of each complex. The root mean square deviation (RMSD) was used to monitor conformational fluctuations, where a steady curve indicates that the system has reached equilibrium [36,37]. As shown in Figure 2A, the RMSD trajectory of the ganoderenic acid A–7BW1 complex equilibrated after 11.58 ns, yielding a mean RMSD value of 0.43 ± 0.026 nm. Similarly, the ganoderic acid DM–7BW1 complex attained equilibrium at 34.24 ns, fluctuating around a mean RMSD value of 0.43 ± 0.027 nm. In contrast, the ganoderic acid B–7BW1 complex exhibited pronounced fluctuations during the initial stages, eventually reaching conformational equilibrium at 73.69 ns with a mean RMSD of 0.29 ± 0.026 nm. In all cases, the systems reached conformational equilibrium within 100 ns, indicating that the ligands stably bound near the SRD5A2 active pocket. Notably, the ganoderic acid DM complex exhibited the most stable profile, suggesting strong binding stability.

Residue fluctuations within the protein backbone were assessed via root-mean-square fluctuation (RMSF) analysis. While higher RMSF scores indicate greater local flexibility, lower values reflect rigidity and stable conformations [38]. In the ganoderic acid DM-bound system, despite localized flexibility in residues 31–43 and 70–75, the RMSF values of the key ligand-binding residues (Leu20, Leu23, Ala24, Val27, Lys29, Trp53, Gln56, Arg94, Tyr107, Leu111, Ile112, Arg114, Gly115, Phe118, Phe219, Phe223, and Arg227) all remained below 0.19 nm (Figure 2B). These observations suggest that the binding pocket was rigid and stable, facilitating stable binding of ganoderic acid DM to SRD5A2. A comparable trend was observed in the ganoderenic acid A system: all residues involved in ligand interactions showed RMSF values below 0.13 nm, ensuring stability at the binding site. While the ganoderic acid B system displayed large fluctuations in several regions (explaining its higher overall RMSD), its key interacting residues remained below 0.13 nm. This confirms the structural integrity of the binding pocket for all tested triterpenoids.

The radius of gyration (Rg) was calculated to evaluate the global compactness and stability of the protein. Lower Rg values reflect a highly compact conformation, whereas higher values indicate an expanded structure [39]. Consistent with the RMSD and RMSF results, the Rg profiles remained steady throughout the simulation, with values fluctuating within a narrow range of 1.90–2.04 nm (Figure 2C). These steady profiles demonstrate that triterpenoids bind to SRD5A2 without causing major unfolding or structural changes, further supporting the stable protein–ligand interactions. As hydrogen bonds are key to stabilizing protein–ligand complexes, we evaluated them to explain the stability observed in RMSD and Rg analyses. As depicted in Figure 2D, the number of H-bonds in the ganoderic acid B–7BW1 complex stabilized after 23 ns, fluctuating steadily between 3 and 6 for the rest of the simulation. Furthermore, the ganoderic acid DM and ganoderenic acid A complexes maintained stable hydrogen-bonding throughout the simulation, with H-bond numbers ranging from 3–6 and 2–5, respectively. These findings demonstrate that all three triterpenoids form stable H-bonding with 7BW1 receptor, directly stabilizing the complexes.

**Figure 3:**
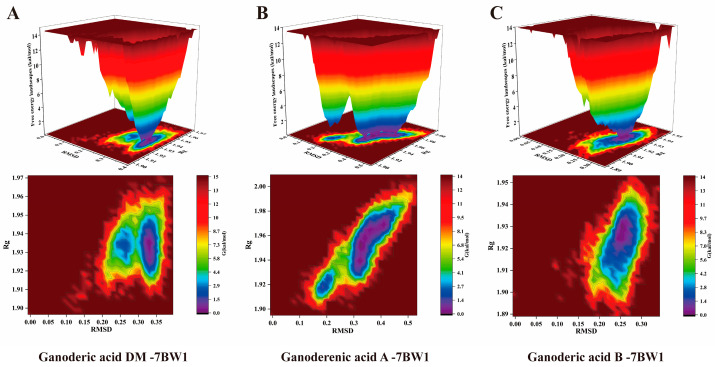
(**A**–**C**): 3D and 2D of Gibbs free energy landscape for ganoderic acid DM-7BW1 (**A**), ganoderenic acid A-7BW1 (**B**), and ganoderic acid B-7BW1 (**C**) complexes; 7BW1: the crystal structure of SRD5A2.

A free energy landscape (FEL) was generated using RMSD and Rg, whereby the most stable, minimum-energy conformations were accurately identified. Theoretically, weak or unstable interactions yield a rough free energy landscape with multiple scattered basins, whereas robust interactions show as a single, well-defined minimum-energy basin [37]. As illustrated in Figure 3A–C, the FELs of all three complexes feature a single, deeply focused low-energy basin (marked by the dark blue/purple regions). The absence of scattered unstable states clearly confirms the formation of stable complexes, validating both the initial docking poses and the structural integrity maintained during the simulations.

Ultimately, the MM-PBSA method was used to calculate the binding free energies (Δ*G*_Bind_) (Table 3). Ganoderic acid DM exhibited the strongest affinity with a value of −52.16 ± 4.40 kJ/mol, followed by ganoderenic acid A (−41.40 ± 5.57 kJ/mol) and ganoderic acid B (−23.42 ± 8.35 kJ/mol). As evidenced by the energy decomposition, the stability of these complexes was primarily driven by electrostatic (Δ*E*_elec_) and van der Waals forces (Δ*E*_vdW_). Subsequently, per-residue energy decomposition was conducted to determine key contributors (ΔG < −5 kJ/mol) and supporting residues (−1 to −5 kJ/mol) (Figure S5A–C). In the ganoderic acid DM complex, Arg227 (−7.507 kJ/mol) was identified as the primary hotspot residue, while Leu20, Lys29, Arg94, Tyr107, Phe118, and Phe223 provide supporting stabilization. Arg114 (−7.599 kJ/mol) emerged as the central hotspot for ganoderenic acid A, while Arg94 (−11.377 kJ/mol) and Arg171 (−8.707 kJ/mol) constituted the core hotpots for ganoderic acid B.

#### 3.4.2. Averaged Weak Interactions Analysis

**Figure 4:**
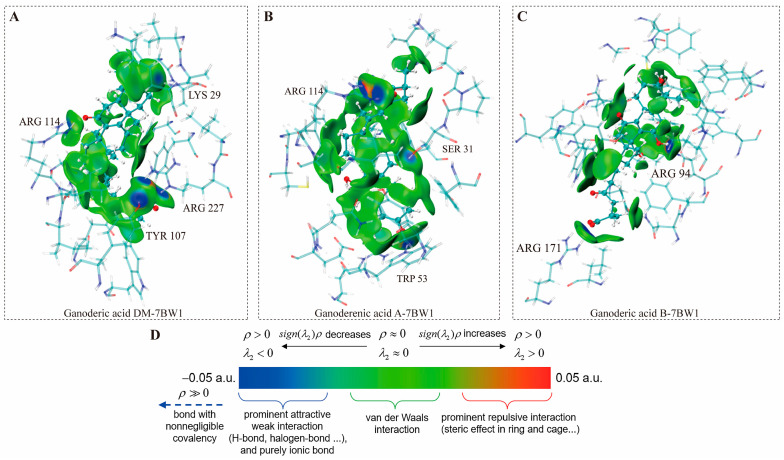
(**A**–**C**): amIGM isosurfaces of weak interactions between the three triterpenoids and 7BW1. ganoderic acid DM (**A**), ganoderenic acid A (**B**), and ganoderic acid B (**C**). (**D**) The standard interpretation of the color coding scheme for the *sign (λ*_2_*)ρ* scalar function.

To examine the weak noncovalent interactions (NCIs) between the three triterpenoids and SRD5A2, amIGM analysis was performed on stable MD snapshots using Multiwfn. The *sign(λ_2_)ρ* color scheme (Figure 4D) represents strong attractions in blue (such as hydrogen bonds), weak vdW interactions in green, and strong repulsion in red [32,40]. Consistent with this color scheme, Figure 4A–C reveal extensive green isosurfaces at the binding interfaces, establishing vdW forces as the dominant NCI factor. Notably, the ganoderic acid DM–7BW1 complex (Figure 4A), exhibited four clear blue isosurfaces (interacting with Lys29, Tyr107, Arg114, and Arg227), reflecting the most pronounced H-bonding among the complexes. Conversely, the ganoderenic acid A–7BW1 complex (Figure 4B) featured a binding interface predominantly covered by green isosurfaces, with localized blue hydrogen-bonding regions interacting with Ser31, Trp53, and Arg114. The ganoderic acid B–7BW1 complex exhibited the smallest surface coverage at the binding interface (Figure 4C). Blue hydrogen-bonding isosurfaces were exclusively detected at Arg94 and Arg171, while all other regions were dominated by green isosurfaces, indicating that its NCIs are almost entirely vdW-driven. Together, these NCI features strongly support the binding modes and energy decomposition results from the MD simulations, further elucidating the molecular mechanism behind the affinities of these triterpenoids for SRD5A2.

### 3.5. Content Determination of the Target Active Compounds

UPLC-Q-TOF-MS/MS was employed to quantify the active triterpenoids. Its high sensitivity and ease of use effectively overcome the measurement errors of co-eluting components common in conventional HPLC [41]. Specifically, active triterpenoids (P8, P9, and P21) were selected due to their VIP > 1 and their positive correlation with SRD5A2 inhibitory activity. As presented in Table 4, varieties H3 and H4 accumulated significantly higher levels of these bioactive components at S1 than S2 and S3. Among these, H3S1 exhibited the highest concentration, followed closely by H4S1. In contrast, maximum accumulation in H1 and H2 occurred at S2. This chemical profile closely mirrored the biological data, as samples with high triterpenoid content produced stronger SRD5A2 inhibition. (Data for the other triterpenoids are detailed in Table S3.) In conclusion, this work elucidates the relationship between specific triterpenoid profiles and in vitro SRD5A2 inhibition, offering a targeted reference for evaluating the functional potential of different *G. lucidum* varieties.

### 3.6. Quality Evaluation of G. lucidum Based on SQFM

**Figure 5:**
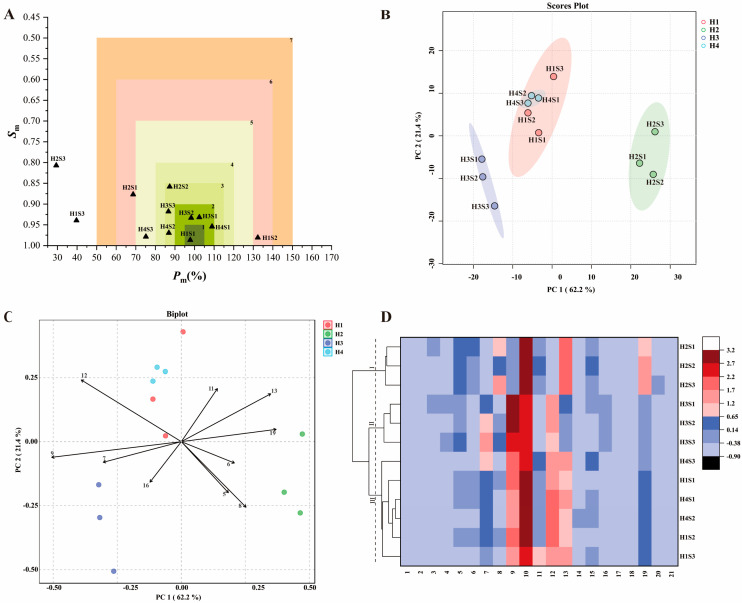
(**A**): Gate diagram-based hierarchical classification results; (**B**–**D**): PCA score plot (**B**), biplot (**C**), and Hierarchical clustering heatmap (**D**) of H1–H4 across different growth stages.

The SQFM was employed using RFP as the standard to conduct a comprehensive qualitative and quantitative evaluation of *G. lucidum* quality. Table 2 presents the calculated *S*_m_, *P*_m_, and *α* values for all samples. Overall, each sample yielded *S*_m_ values > 0.80 and *α* values < 0.12, indicating consistent chemical profiles. In contrast, the *P*_m_ values exhibited a broad range (29.4% to 132.3%), revealing quantitative variations in triterpenoid content among the investigated samples. As depicted in Figure 5A and Table 2, H2S3 exhibited the lowest *S*_m_ and *P*_m_ values, indicating a marked deviation from the RFP. Statistical analysis confirmed that these lower *P*_m_ values corresponded directly to the reduced contents of target compounds in its common peaks. Conversely, H1S1, H3S1, H3S2, and H4S1 displayed *S*_m_ and *P*_m_ values closely approximating the RFP, indicating a high degree of chemical consistency. The H3 variety uniformly maintained high chemical stability across the monitored stages, with no samples falling below Grade 3. As reflected in the comprehensive grading results (Figure 5A), samples collected at the S1 and S2 growth stages exhibited higher quantitative similarity than those harvested at the S3. This temporal trend highlights the combined utility of *S*_m_ and *P*_m_ for both qualitative and quantitative assessments. Integrating these results with our preliminary study, H3S1 exhibited the most potent SRD5A2 inhibitory activity among the samples with high triterpenoid content, followed by H4S1. Ultimately, this approach provides a practical, chromatography-based metric for monitoring the chemical consistency of *G. lucidum* raw materials.

### 3.7. Chemometric Analysis

#### 3.7.1. Principal Component Analysis (PCA)

To understand the chemical basis for the quantitative variations revealed by SQFM, an unsupervised PCA was executed utilizing the MetaboAnalyst platform. Based on a data matrix comprising 12 observations and 21 chemical components, the first two principal components accounted for a cumulative variance of 83.6% (PC1: 62.2%; PC2: 21.4%, as depicted in Figure 5B,C). This high explanatory capacity verifies the model’s reliability in elucidating the metabolic diversity of the dataset. As depicted in Figure 5B, the samples separated into three distinct clusters. Throughout all growth stages, varieties H1 and H4 clustered in the positive region of PC2, while strain H2 separated exclusively along the positive axis of PC1. In contrast, the H3 variety cleanly segregated into the third quadrant, exhibiting negative scores on both principal components. Furthermore, the clustering patterns of the H2 and H3 varieties across growth stages were highly consistent with the results of the SQFM analysis. Loadings vectors (Figure 5C) identify the specific triterpenoids driving this differentiation. The H2 variety is distinguished by an accumulation of highly oxygenated triterpenoids, notably P13, and P19, owing to their strong positive PC1 loadings. For strain H3, P7, P9, and P16 act as signature markers pointing toward the third quadrant. Conversely, driven by the negative PC1 loading of P12, H1 and H4 clustered along PC2. Collectively, these results validate the reliability of the SQFM strategy as a complementary mathematical tool for assessing the chemical consistency of *G. lucidum*.

#### 3.7.2. Hierarchical Clustering Heatmap Analysis

To further visualize these chemical differences between varieties and validate the PCA loadings, a hierarchical clustering analysis (HCA) heatmap was generated in Origin 2024 (average linkage method, Manhattan distance). As shown in Figure 5D, the row clustering corroborates the previously observed PCA separation, yielding three distinct clades: Clade I (H2), Clade II (H3), and Clade III (H1 and H4). This branching pattern visually confirms the chemical similarity shared between H1 and H4. The sub-clustering within Clades I and II matches the temporal trends in *P*_m_ values. Specifically, S1 and S2 samples grouped together, while the S3 samples remained distinct. Column analysis of the heatmap visually validates the aforementioned marker compounds. The high amounts of oxygenated P13/P19 in H2 and P7/P9/P16 in H3, clearly differ from the moderate levels of the unsaturated P12 in Clade III. This reverse relationship reflects metabolic change linked to genetic differences between varieties. Finally, the universal high abundance of ganoderic acid A (P10) in all samples shows its role as a basic metabolite in *G. lucidum*. In summary, the combined use of SQFM, PCA, and hierarchical clustering heatmap establishes a robust chemometric workflow for characterizing the chemical diversity and monitoring the chemical consistency of *G. lucidum*.

## 4. Discussion

In this study, an integrated evaluation strategy was constructed by correlating chromatographic profiles with bioactivity, alongside computational chemistry (MD and aNCI), SQFM, and chemometrics. Overcoming the limitations of traditional single-method approaches, this strategy combines multiple analytical techniques to provide a more comprehensive assessment. Ultimately, it spans from active triterpenoid identification to chemical consistency monitoring, thereby providing a practical methodological framework for the systematic quality control of edible–medicinal fungi like *G. lucidum*.

Regarding the targeted bioactivity, an OPLS regression model identified seven characteristic peaks highly correlated with SRD5A2 inhibition (Figure 1C,D). Supported by in vitro assays (Figure S4 and Table S2) and confirmed by recent complex mixture analyses [42], this correlation-based approach not only aids in screening potential inhibitors but also helps overcome conventional isolation bottlenecks [9,10]. Furthermore, validating the robustness of OPLS regression model, ganoderic acid DM emerged as the potent inhibitor among the three identified compounds, a finding highly consistent with previous literature [4]. Crucially, integrating MD simulations and aNCI analysis advanced the macroscopic correlation to a microscopic mechanistic level. These models revealed that active triterpenoids inhibit SRD5A2 primarily through stable hydrogen bonds and hydrophobic interactions (Table 3 and Figure 4). Aligning closely with previous structural studies [43], these results provide mechanistic insights into the SRD5A2 inhibitory potential of specific *G. lucidum* triterpenoids. Furthermore, the approach supports a computational paradigm for natural inhibitor discovery without exhaustive traditional isolation, which is a strategy increasingly validated in the study of bioactive food components [6,44].

Parallel to the bioactivity assessment, the chemical profiling utilized SQFM, PCA, and hierarchical clustering heatmaps to address the quantitative limitations of conventional chromatographic fingerprinting [20,45]. This systematic profiling revealed the metabolic fluctuations across varieties and growth stages. Notably, within the H1, H3, and H4 varieties, the targeted triterpenoid content consistently peaked at the initial fruiting body formation stage (S1) and was lowest at the spore-release stage (S3) (Table 2). As corroborated by recent studies, this S1 optimum is primarily driven by the differential expression of triterpenoid biosynthetic enzymes [46,47,48]. Consequently, early harvesting of superior varieties (like H3) maximizes functional ingredient yield, offering suitable *G. lucidum* materials for the development of BPH-related functional foods.

In summary, correlating targeted bioactivity with chemical profiles ensures functional relevance, while SQFM provides quantitative metrics for macroscopic batch-to-batch consistency. This integrated strategy effectively offers a practical solution to long-standing industrial quality control bottlenecks for *G. lucidum*. Future research will incorporate transcriptomics to elucidate the underlying biosynthetic pathways driving the H3 variety, laying a foundation for the standardized application of functional foods.

## 5. Conclusions

In conclusion, this study integrated evaluation strategy combining targeted bioactivity correlation and chemical profiling for *G. lucidum*. Through this approach, we identified seven characteristic peaks correlated with in vitro SRD5A2 inhibition, characterizing ganoderic acids DM and B, as well as ganoderenic acid A, as key bioactive triterpenoids. In vitro validations and mechanistic models confirmed their SRD5A2 inhibitory potential, revealing that hydrogen bonds and hydrophobic interactions act as the primary drivers for the robust SRD5A2 inhibition. Furthermore, chemometric analysis elucidated the ontogenetic variations in triterpenoid content, while the SQFM calculations provided practical metrics for assessing chemical consistency. Notably, the H3S1 sample exhibited both the highest targeted active ingredient content and potent SRD5A2 inhibition. Ultimately, this strategy offers a practical reference to support the standardized application and future development of *G. lucidum*-based functional foods.

## Figures and Tables

**Table 1:** Compounds identified in *G. luciudm* samples H1−H4 at three fruiting body growth stages (S1, S2, and S3) by UPLC-ESI-MS/MS.

NO.	t*_R_* (Min)	[M − H]		MS/MS	Formula	Identification
		Measured Value	Error			
**1**	4.11	533.3106	−2.6	301.1809, 195.1026, 79.0544	C_30_H_46_O_8_	Ganoderic acid L
**2**	4.28	475.2698	−0.6	265.1444, 149.0968, 123.0803	C_27_H_40_O_7_	Lucidenic acid G
**3**	4.31	529.2802	−0.8	475.2693, 305.1753, 149.0968	C_30_H_44_O_9_	(3*α*,5*β*,7*α*,10*α*,12*α*,13*α*,14*β*,17*α*,20*R*)-3,7,12,20-Tetrahydroxy-11,15,23-trioxolanost-8-en-26-oic acid
**4**	4.67	527.2646	−0.9	453.2283, 317.1754, 129.0556	C_30_H_40_O_8_	Elfvingic acid B
**5**	4.93	513.2857	−0.1	427.2136, 287.1639, 79.0542	C_30_H_44_O_8_	(3*β*,7*β*,12*β*,17*α*,20*S*)-3,7,12-Trihydroxy-11,15,23-trioxolanost-8-en-26-oic acid
**6**	4.97	377.1966	−0.9	319.1903, 287.1640	C_21_H_30_O_6_	Ganomycin J
**7**	5.10	529.2808	0.1	399.2151, 301.1803, 129.0552	C_30_H_44_O_9_	20-Hydroxyganoderic acid G
**8**	5.95	457.2588	−1.7	439.2471, 303.1972, 111.0444	C_27_H_38_O_6_	Ganolactone B
**9**	6.00	531.2959	−0.8	483.2374, 303.1972, 129.0552	C_30_H_44_O_8_	Ganoderic acid I
**10**	6.71	515.3010	−0.8	441.2644, 249.1500, 73.0284	C_30_H_44_O_7_	Ganoderic acid DF
**11**	7.24	507.2961	−0.4	461.2908, 287.1642, 155.0715	C_27_H_42_O_6_	Lucidenic acid M
**12**	7.50	515.3014	−0.1	443.2056, 193.0866, 79.0542	C_30_H_44_O_7_	Ganoderenic acid C
**13**	8.30	475.2707	1.2	427.2122, 303.1962, 109.0651	C_27_H_40_O_7_	Lucidenic acid C
**14**	8.61	571.2911	−0.2	455.2428, 177.0917, 109.0653	C_32_H_44_O_9_	(15*β*,25*S*)-28-Acetoxy-15-hydroxy-3,7,11,23-Tetraoxolanost-8-en-26-oic acid
**15**	8.71	517.3173	0.5	499.3062, 287.1648, 151.1122	C_30_H_46_O_7_	Ganoderic acid C2
**16**	9.36	531.2957	−1.1	469.2953, 287.1658, 129.0551	C_30_H_44_O_8_	(3*β*,7*β*,12*β*,23*S*,24*E*)-3,7,12,23-tetrahydroxy-11,15-dioxolanosta-8,24-dien-26-oic acid
**17**	9.50	529.2801	−1	511.2678, 303.1952, 129.0554	C_30_H_42_O_8_	(3*β*,7*β*,12*β*,20*Z*)-3,7,12-Trihydroxy-11,15,23-trioxolanosta-8,20(22)-dien-26-oic acid
**18**	9.96	459.2755	0.5	343.113, 249.1495, 59.0126	C_27_H_40_O_6_	Lucidenic acid N
**19**	10.47	511.2694	−1.4	437.2336, 303.1602, 189.1287	C_30_H_42_O_8_	Ganoderic acid C6
**20**	11.41	529.2805	−0.3	317.1736, 300.1696, 73.0286	C_30_H_42_O_8_	12-Hydroxyganoderic acid D
**21**	11.47	513.2852	−1.1	265.1446, 207.1388, 149.0968	C_30_H_44_O_8_	Ganoderic acid G
**22**	11.94	513.2855	−0.6	495.2749, 249.1495, 147.0811	C_30_H_42_O_7_	Ganoderenic acid B
**23**	12.02	515.3003	−2.1	499.3028, 285.1496, 149.0603	C_30_H_44_O_7_	Ganoderic acid gamma
**24**	12.36	529.2805	−0.4	399.2172, 301.1800, 129.0552	C_30_H_42_O_8_	Ganoderic acid N
**25**	12.63	497.2909	0.2	303.1966, 249.1496, 79.0544	C_30_H_44_O_7_	Ganoderic acid B
**26**	12.91	511.2696	−0.9	495.2764, 301.1796, 147.0813	C_30_H_40_O_7_	Ganoderenic acid H
**27**	13.51	513.2857	−0.2	473.2541, 301.1808, 195.1020	C_30_H_42_O_7_	Ganoderic acid AM1
**28**	13.55	515.2657	1.4	473.2541, 301.1808, 195.1020	C_29_H_40_O_8_	Lucidenic acid E
**29**	13.93	571.2917	0.9	553.2799, 303.1965, 129.0552	C_32_H_44_O_9_	12*β*-Acetoxy-3*β*,7*β*-dihydroxy-11,15,23-trioxo-lanost-8,16-dien-26-oic acid
**30**	14.31	513.2860	0.5	301.1801, 193.0867, 79.0544	C_30_H_42_O_7_	Ganoderenic acid A
**31**	14.35	555.2964	0.2	513.2856, 301.1801, 79.0544	C_32_H_46_O_9_	Ganoderic acid K
**32**	14.46	473.2547	0.4	425.1963, 407.1849, 284.1397	C_27_H_38_O_7_	Lucidenic acid B
**33**	14.63	505.2807	0.1	285.1492, 149.0605	C_27_H_40_O_6_	Chol-8-en-24-oic acid, 7,15-dihydroxy-4,4,14-trimethyl-3,11-dioxo-, (5*α*)-
**34**	14.78	513.2856	−0.3	193.0869, 79.0542	C_30_H_42_O_7_	(15*α*,25*R*)-15-Hydroxy-3,7,11,23-tetraoxolanost-8-en-26-oic acid
**35**	14.92	527.2647	−0.6	435.2170, 301.1438, 189.1281	C_30_H_40_O_8_	(7*β*,20*E*)-2,7-Dihydroxy-3,11,15,23-tetraoxolanosta-8,20(22)-dien-26-oic acid
**36**	15.19	529.2805	−0.4	511.2687, 299.1646, 137.0601	C_30_H_42_O_8_	Ganoderic acid C6 isomer
**37**	15.28	553.2803	−0.8	467.2794, 284.1405, 59.0125	C_32_H_44_O_9_	(12*β*,20*S*)-12-Acetoxy-3-hydroxy-7,11,15,23-tetraoxolanost-8-en-26-oic acid
**38**	15.35	527.2643	−1.5	447.2527, 299.1645, 263.1285	C_30_H_40_O_8_	Ganoderenic acid E
**39**	15.47	569.2747	−1.5	509.2544, 479.2064, 302.1519	C_32_H_42_O_9_	Ganoleuconin F
**40**	15.71	515.3012	−0.5	497.2907, 285.1496, 149.0604	C_30_H_44_O_7_	Ganoderic acid A
**41**	15.95	511.2699	−0.4	437.2333, 301.1795, 149.0603	C_30_H_42_O_8_	Methyl lucidenate E2
**42**	15.99	553.2804	−0.6	437.2333, 301.1795, 149.0603	C_32_H_44_O_9_	Ganoderic acid H
**43**	16.21	501.3218	−0.8	439.3224, 287.2013	C_30_H_46_O_6_	Ganolucidic acid B
**44**	16.52	455.2432	−1.6	351.2322, 335.2008, 149.0601	C_27_H_36_O_6_	Dehydrolucidenic acid A
**45**	16.64	499.3062	−0.7	437.3025, 287.2013, 85.0284	C_30_H_44_O_6_	Ganolucidic acid D
**46**	16.81	509.2541	−0.8	435.2177, 301.1441, 59.0127	C_30_H_40_O_8_	Deacetyl ganoderic acid F
**47**	17.00	555.2960	−0.6	515.3011, 423.2541, 129.0553	C_32_H_46_O_9_	Ganoderic acid K isomer
**48**	17.02	515.3007	−1.3	423.2541, 303.1959	C_30_H_44_O_7_	(3*β*,5xi,7*β*,25*R*)-3,7-Dihydroxy-11,15,23-trioxolanost-8-en-26-oic acid
**49**	17.17	511.2700	−0.2	299.1651, 263.1288, 137.0604	C_30_H_42_O_8_	Ganoderic acid D2
**50**	17.18	457.2598	0.6	299.1651, 263.1288, 137.0604	C_27_H_38_O_6_	Lucidenic acid A
**51**	17.48	515.3016	0.3	497.2915, 301.1805, 79.0543	C_30_H_44_O_7_	Ganoleuconin B
**52**	18.04	511.2703	0.4	493.2592, 449.2691, 149.0602	C_30_H_40_O_7_	Ganoderenic acid D
**53**	18.77	511.2704	0.6	493.2595, 449.2702, 147.0809	C_30_H_40_O_7_	(7*β*)-7-Hydroxy-3,11,15,23-tetraoxolanosta-8,16-dien-26-oic acid
**54**	18.92	495.2757	1	301.1807, 149.0604, 97.0650	C_30_H_42_O_7_	Ganoderic acid D
**55**	19.51	509.2546	0.3	299.1652, 149.0606	C_30_H_38_O_7_	Ganoderenic acid F
**56**	19.65	569.2759	0.5	551.2650, 465.2634, 285.1487, 99.0445	C_32_H_42_O_9_	(7*β*,12*β*,20*Z*)-12-Acetoxy-7-hydroxy-3,11,15,23-tetraoxolanosta-8,20(22)-dien-26-oic acid
**57**	19.79	497.2908	−0.2	287.2012, 149.0957	C_30_H_42_O_6_	Lanosta-8,20(22)-dien-26-oic acid, 15-hydroxy-3,11,23-trioxo-, (15*α*,20*Z*)-
**58**	19.86	513.2498	0.7	441.1918, 340.1316, 149.0957	C_29_H_38_O_8_	Lucidenic acid D
**59**	20.03	501.3225	0.8	287.1649, 249.1508	C_30_H_46_O_6_	Rel-2*α*,3*α*,23-trihydroxy-19-oxo-18,19-seco-urs-11,13(18)-dien-28-oic acid
**60**	20.15	511.2703	0.4	419.2226, 299.1648, 149.0603	C_30_H_40_O_7_	Ganoderenic acid H isomer
**61**	20.18	553.2803	−0.7	299.1648, 285.1494, 149.0603	C_32_H_44_O_9_	Ganodernoid G
**62**	20.73	499.3065	0	437.3058, 285.1860, 151.1127	C_30_H_44_O_6_	Ganolucidic acid A
**63**	20.81	553.2797	−1.8	449.2685, 299.1646, 59.0121	C_32_H_44_O_9_	Isomer of 12*β*-acetoxy-3*β*,7*β*-dihydroxy-11,15,23-trioxo-lanost-8,16-dien-26-oic acid
**64**	21.02	567.2596	−0.6	507.2394, 477.1907	C_32_H_40_O_9_	Ganodernoid D
**65**	21.09	513.2847	−2	497.2921, 287.2027, 149.0602	C_30_H_42_O_7_	Ganoderic acid Z
**66**	21.28	657.2923	1	553.2805, 479.2439, 345.1799	C_35_H_46_O_12_	12-Acetoxy-3-(2-carboxyacetoxy)-7,11,15,23-tetraoxolanost-8-en-26-oic acid
**67**	21.45	551.2650	0	435.2178, 301.1440, 189.1285	C_32_H_42_O_9_	Ganoderic acid F
**68**	21.69	513.2854	−0.7	421.2381, 301.1803, 285.1492	C_30_H_42_O_7_	Ganoderic acid J
**69**	22.74	555.2588	−2.1	435.2141, 287.1985	C_30_H_38_O_7_	(7*β*,20*E*)-7-Hydroxy-3,11,15,23-tetraoxolanosta-8,16,20(22)-trien-26-oic acid
**70**	23.03	533.3484	0	283.1368, 99.0443	C_31_H_50_O_7_	Methyl 4,23,29-trihydroxy-3,4-seco-olean-12-en-3-oate-28-oic acid
**71**	23.06	507.2386	−0.4	433.2018, 283.1368, 197.0273	C_30_H_36_O_7_	Tanariflavanone A
**72**	23.41	499.3061	−0.8	285.1479, 247.1349, 149.0601	C_30_H_44_O_6_	Ganoderic acid GS-2
**73**	23.61	485.3274	0.2	289.2171, 99.0447	C_30_H_46_O_5_	Ganoderic acid XL3
**74**	24.37	499.3061	−0.8	435.2886, 297.1527, 133.0655	C_30_H_44_O_6_	(3*β*,7*β*,24*E*)-3,7-Dihydroxy-11,15-dioxolanosta-8,24-dien-26-oic acid
**75**	24.57	555.2946	−3.2	343.1913, 283.1708, 149.0594	C_32_H_46_O_9_	Ganoderic acid *α* isomer
**76**	25.52	595.2906	−1.1	479.2428, 311.1679, 165.0917	C_34_H_46_O_10_	(4*β*,5*β*,6*α*,22*R*)-5-Hydroxy-1,26-dioxo-22,26-epoxyergosta-2,24-dien-4,6,27-triyl-triacetat
**77**	25.65	509.2528	−3.3	297.1530, 119.0498	C_30_H_40_O_8_	Applanoxidic acid G
**78**	25.66	483.3108	−1.7	297.1530, 183.0119, 119.0498	C_30_H_44_O_5_	Ganolucidic acid E
**79**	26.02	483.3112	−0.8	345.2063, 271.1705	C_30_H_44_O_5_	(11*α*,24*E*)-11-Hydroxy-3,7-dioxolanosta-8,24-dien-26-oic acid
**80**	26.32	343.1909	−1.8	183.0118, 119.0497	C_21_H_28_O_4_	Ganomycin B
**81**	26.78	527.3370	−1.6	485.3301, 311.1681	C_32_H_48_O_6_	Ganoderic acid DM isomer
**82**	27.23	483.3117	0.2	325.1842, 271.1684, 159.0446	C_30_H_44_O_5_	(24*E*)-3-Oxolanosta-8,24-diene-21,26-dioic acid
**83**	28.10	295.2274	−1.5	243.8996	C_18_H_32_O_3_	9-hydroxy-10*E*,12*E*-octadecadienoic acid
**84**	28.39	295.2273	−1.9	197.0275, 116.9279	C_18_H_32_O_3_	9-HODE
**85**	28.91	525.3214	−1.4	483.3111, 287.2017, 99.0445	C_32_H_46_O_6_	(22*S*,24*E*)-22-Acetoxy-3,7-dioxolanosta-8,24-dien-26-oic acid
**86**	29.46	517.3530	−1	311.1683, 183.0121, 119.0500	C_30_H_48_O_4_	Ganodermanontriol
**87**	29.94	297.2432	−1	114.0186	C_18_H_34_O_3_	18-Hydroxy-9-octadecenoic acid
**88**	30.58	295.2274	−1.7	279.2324, 141.0921	C_18_H_34_O_4_	Octadecanedioic acid
**89**	30.84	295.2274	−1.4	183.1012, 116.9279	C_18_H_34_O_4_	(8*E*)-7,10-Dihydroxy-8-octadecenoic acid
**90**	31.02	467.3158	−1.8	423.3264, 285.1856	C_30_H_44_O_4_	Ganoderic acid DM
**91**	31.27	467.3162	−1	255.1754, 119.0498	C_30_H_44_O_4_	Ganoderic acid TR
**92**	32.17	467.3165	−0.5	353.2143, 99.0447	C_30_H_44_O_4_	16*α*-Hydroxy-3-oxolanosta-7,9(11),24-triene-21-oic acid
**93**	37.25	281.2476	−3.6	183.0119	C_18_H_34_O_2_	6-Octadecenoic acid

**Table 3:** Energy components of ganoderic acid DM-7BW1, ganoderenic acid A-7BW1, and ganoderic acid B-7BW1 complexes (kJ/mol).

Energy Components	Ganoderic Acid DM	Ganoderenic Acid A	Ganoderic Acid B
Δ*E*_vdW_	−47.04 ± 3.63	−50.58 ± 3.27	−50.93 ± 3.56
Δ*E*_elec_	−308.99 ± 10.91	−310.02 ± 12.96	−314.03 ± 17.21
Δ*E*_PB_	308.93 ± 8.72	324.5 ± 10.62	347.29 ± 17.39
Δ*E*_NPOLAR_	−5.06 ± 0.13	−5.3 ± 0.14	−5.75 ± 0.09
Δ*G*_GAS_	−356.03 ± 10.57	−360.6 ± 12.91	−364.97 ± 16.44
Δ*G*_SOLV_	303.87 ± 8.66	319.2 ± 10.64	341.54 ± 17.36
Δ*G*_Bind_	−52.16 ± 4.40	−41.4 ± 5.57	−23.42 ± 8.35

**Table 4:** Contents of ganoderic acid DM, ganoderenic acid A, and ganoderic acid B in H1–H4 at three fruiting body growth stages (mg/g).

Strain		Ganoderic Acid DM	Ganoderenic Acid A	Ganoderic Acid B	Total
H1	S1	0.083 ± 0.0012	0.536 ± 0.0217	0.454 ± 0.0148	1.073 ± 0.0377
	S2	0.074 ± 0.0018	0.699 ± 0.0092	0.491 ± 0.0168	1.264 ± 0.0242
	S3	0.056 ± 0.0005	0.328 ± 0.025	0.089 ± 0.0045	0.473 ± 0.02
H2	S1	0.098 ± 0.0003	0.13 ± 0.0011	1.001 ± 0.0788	1.229 ± 0.078
	S2	0.103 ± 0.0047	0.208 ± 0.0203	1.099 ± 0.0003	1.411 ± 0.0153
	S3	0.071 ± 0.0016	0.095 ± 0.0241	0.445 ± 0.1168	0.612 ± 0.1393
H3	S1	0.373 ± 0.0528	1.301 ± 0.0356	0.553 ± 0.0194	2.227 ± 0.1078
	S2	0.193 ± 0.0283	0.807 ± 0.0393	0.466 ± 0.0031	1.466 ± 0.0707
	S3	0.148 ± 0.012	0.469 ± 0.0463	0.499 ± 0.0045	1.116 ± 0.0538
H4	S1	0.049 ± 0.0058	1.138 ± 0.0219	0.55 ± 0.0399	1.738 ± 0.056
	S2	0.024 ± 0.0004	0.741 ± 0.0179	0.299 ± 0.0122	1.065 ± 0.0053
	S3	0.036 ± 0.0024	0.629 ± 0.131	0.227 ± 0.0154	0.893 ± 0.1488

## Data Availability

The original contributions presented in this study are included in the article/Supplementary Materials. Further inquiries can be directed to the corresponding authors.

## Supplementary Materials

The following supporting information can be downloaded at: https://www.mdpi.com/article/10.3390/foods15122071/s1, Figure S1: *G. lucidum* samples H1–H4 at three fruiting body growth stages; Figure S2: Fingerprint of H1–H4 across different growth phases; Figure S3: HPLC chromatograms of 17 ganoderic acids reference standards; Figure S4: SRD5A2 inhibitory activity of reference standard from H1 to H4 cultivars at three distinct growth stages. Different letters (a–f) above bars in figure indicate significant differences (*p* < 0.05) by Tukey’s test; Figure S5: A, B, and C: Residue energy contribution to the total binding energy in the ganoderic acid DM -7BW1 (A), ganoderenic acid A -7BW1 (B), and ganoderic acid B -7BW1 (C) complexes; Figure S6: Validation plot of the OPLS model using a 200-iteration permutation test. Table S1: Linear regression data, LOD and LOQ of the investigated compounds; Table S2: SRD5A2 inhibition rates (%) of selected reference standards; Table S3: Quantitative results of 30 ganoderic acids in extracts of *G. lucidum* H1–H4 at different growth stages (mg/g).
