# Supplementary material for: Evaluation of Ganoderma lucidum Across Varieties and Growth Stages: Integrating Chromatographic Profiling, Bioactivity Correlation, and In Silico Simulations
Source: Foods. 2026 Jun 8;15(12):2071. doi: 10.3390/foods15122071 (PMC13298459; doi:10.3390/foods15122071)
Supplement: Supplementary file 1 [file foods-15-02071-s001.zip › foods-4333442-supplementary.pdf]

Evaluation of *Ganoderma lucidum* across varieties and growth stages: Integrating chromatographic profiling, bioactivity correlation, and in silico simulations

Xianxian Miao [1], Shuai Zhou [1], Jinyan Wang [1], Jie Feng [1], Zhenhao Li [2], Guoliang, Zhang [2], Na Feng [1,*], Jingsong Zhang [1,*]

[1] *Institute of Edible Fungi, Shanghai Academy of Agricultural Sciences, Shanghai, 201403, China*

[2] *Zhejiang ShouXianGu Botanical Drug Institute, Zhejiang Hangzhou 321200, China*

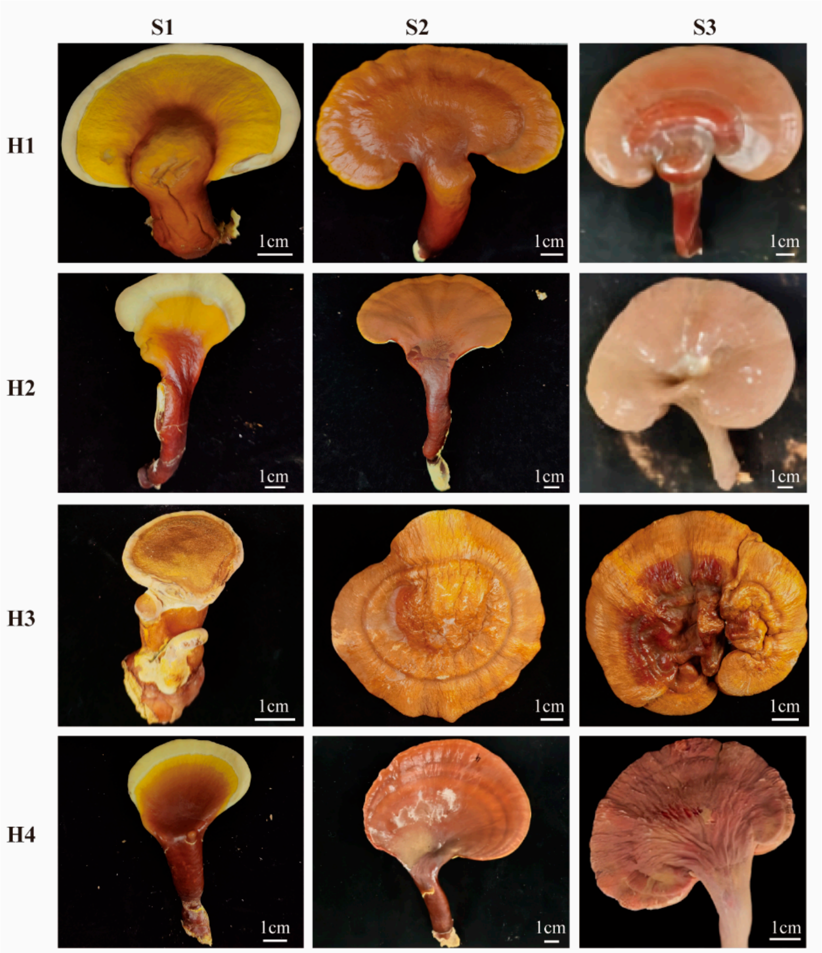

Figure S1. *G. lucidum* samples H1–H4 at three fruiting body growth stages.

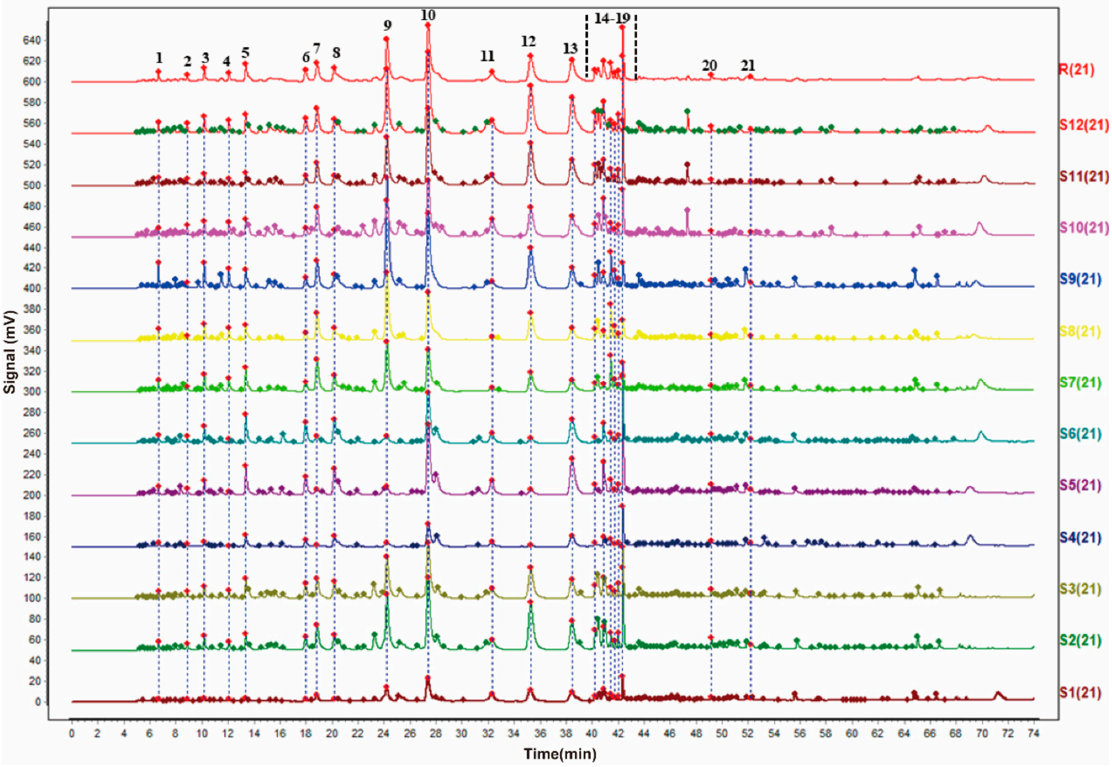

Figure S2. Fingerprint of H1–H4 across different growth phases.

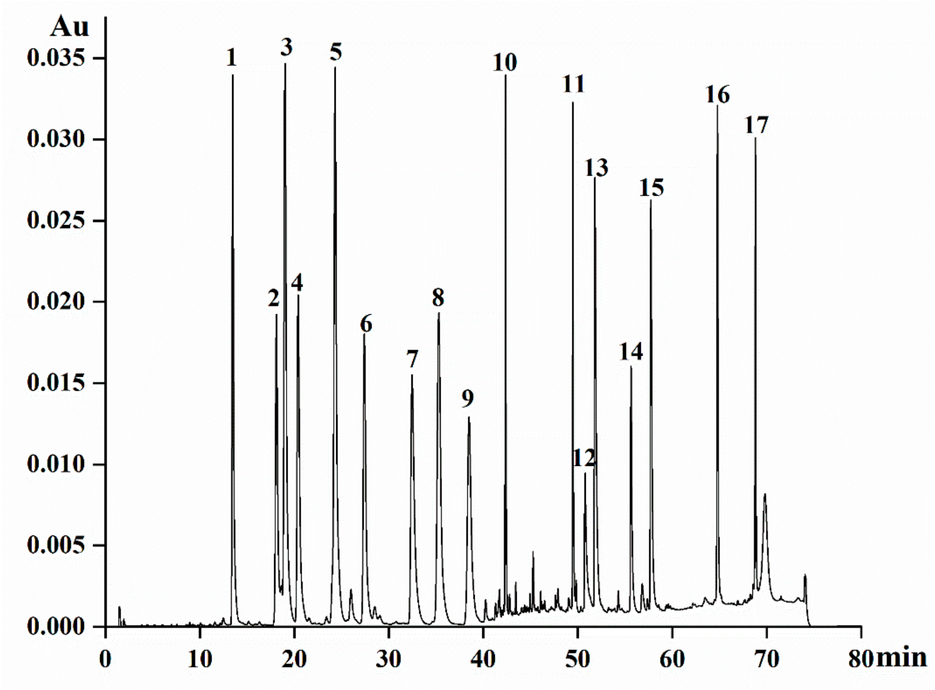

1: Ganoderic acid C2
2: Ganoderic acid G
3: Ganoderenic acid B
4: Ganoderic acid B
5: Ganoderenic acid A
6: Ganoderic acid A
7: Lucidenic acid A
8: Ganoderenic acid D
9: Ganoderic acid D
10: Ganoderic acid F
11: Ganoderenic acid F
12: Ganodermanontriol
13: Ganoderic acid DM
14: Ganoderiol F
15: Ganoderic acid T
16: Ganoderiol B
17: Ganoderal A

Figure S3. HPLC chromatograms of 17 ganoderic acids reference standards.

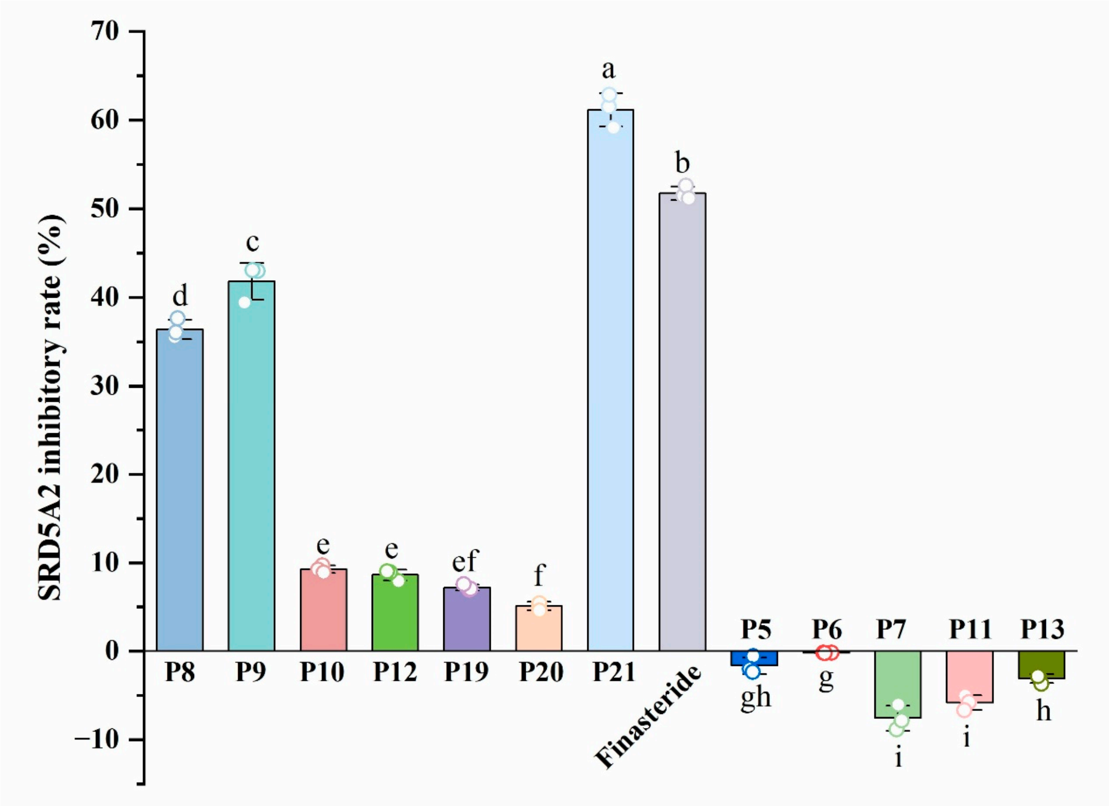

Figure S4. SRD5A2 inhibitory activity of reference standard from H1 to H4 cultivars at three distinct growth stages. Different letters (a–f) above bars in figure indicate significant differences ($p < 0.05$) by Tukey's test.

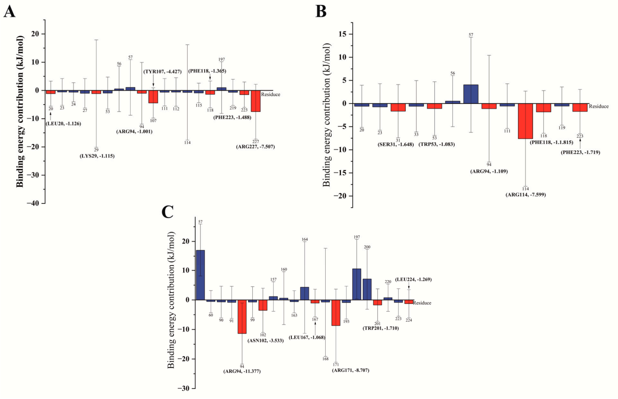

Figure S5. A, B, and C: Residue energy contribution to the total binding energy in the ganoderic acid DM -7BW1 (A), ganoderenic acid A -7BW1 (B), and ganoderic acid B -7BW1 (C) complexes.

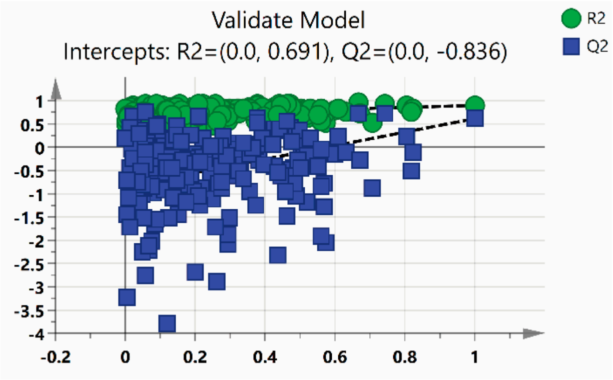

Figure S6. Validation plot of the OPLS model using a 200-iteration permutation test.

Table S1. Linear regression data, LOD and LOQ of the investigated compounds.

standards	Linear regression data			LOD (ng/mL)	LOQ (ng/mL)
	Regressive equation	Test range (ng/mL)	R^2		
Ganoderic acid I	y=38820x+1362	6.0-6000.0	0.9988	0.36	1.09
Ganoderic acid C	y=71881x+38	4.0-4000.0	0.9998	0.07	0.21
Ganoderic acid C2	y=95938x+4756	4.0-4000.0	0.9975	0.30	0.92
Lucidenic acid N	y=125413x-103	4.0-4000.0	0.9995	0.11	0.34
Ganoderic acid C6	y=68616x+2541	4.0-4000.0	0.9981	0.13	0.40
Ganoderic acid G	y=69673x+3941	4.0-4000.0	0.9963	0.48	1.46
Ganoderenic acid B	y=63698x+3902	4.0-4000.0	0.9968	0.18	0.54
Ganoderic acid N	y=38728x+1767	4.0-4000.0	0.9977	0.33	1.00
Ganoderic acid B	y=73915x+4262	4.0-4000.0	0.9963	0.29	0.89
Ganoderenic acid H	y=101795x+23	4.0-4000.0	0.9994	0.03	0.08
Lucidenic acid E	y=66868x+1484	4.0-4000.0	0.9983	0.18	0.54
Ganoderic acid AM1	y=112892x+2297	4.0-4000.0	0.9975	0.10	0.32
Ganoderenic acid A	y=55020x+4091	4.0-4000.0	0.9947	0.51	1.56
Ganoderic acid K	y=79256x+3470	4.0-4000.0	0.9936	0.16	0.48
Lucidenic acid B	y=86076x+78	4.0-4000.0	0.9998	0.06	0.19
Ganoderic acid A	y=96946x+7978	4.0-4000.0	0.9953	0.44	1.33
Ganoderic acid H	y=48061x+3372	4.0-4000.0	0.9945	0.61	1.86
Dehydro-lucidenic acid A	y=102353x+6875	4.0-4000.0	0.9932	0.00	0.01
Deacetyl-ganoderic acid F	y=71491x+1601	4.0-4000.0	0.9978	0.06	0.19
Lucidenic acid A	y=91306x+7688	4.0-4000.0	0.9924	0.32	0.97
Ganoderic acid D2	y=76406x+4922	4.0-4000.0	0.9940	0.43	1.29

Ganoderenic acid D	y=61430x+5288	4.0-4000.0	0.9933	0.37	1.12
Ganoderic acid C1	y=81960x+7082	3.0-3000.0	0.9937	0.27	0.82
Lucidenic acid D	y=93468x+7644	4.0-4000.0	0.9932	0.17	0.53
Ganoderic acid F	y=57413x+10106	4.0-4000.0	0.9837	0.75	2.27
Ganoderic acid J	y=99499x+5843	4.0-4000.0	0.9949	0.06	0.19
Danzhi acid E	y=123262x+3938	4.0-4000.0	0.9952	0.02	0.07
Danodermanontriol	y=48632x+118	4.0-4000.0	0.9990	0.03	0.08
Ganoderic acid DM	y=4129x+81	4.0-4000.0	1.0000	0.14	0.43
Ganoderic acid TR	y=66968x+416	4.0-4000.0	1.0000	0.02	0.05

Table S2. SRD5A2 inhibition rates (%) of selected reference standards.

peaks	Reference standard	Inhibition rates (%)
P8	Ganoderic acid B	36.41 ± 1.1
P9	Ganoderenic acid A	41.82 ± 2.09
P10	Ganoderic acid A	9.26 ± 0.41
P12	Ganoderenic acid D	8.62 ± 0.61
P19	Ganoderic acid F	7.19 ± 0.34
P20	Ganoderenic acid F	5.12 ± 0.46
P21	Ganoderic acid DM	61.16 ± 1.87
PC	Finasteride	51.74 ± 0.77
P5	Ganoderic acid C2	-1.63 ± 0.76
P6	Ganoderic acid G	-0.18 ± 0.02
P7	Ganoderenic acid B	-7.56 ± 1.15
P11	Lucidenic acid A	-5.81 ± 0.67
P13	Ganoderic acid D	-3.08 ± 0.42

Table S3. Quantitative results of 30 ganoderic acids in extracts of *G. lucidum* H1–H4 at different growth stages (mg/g).

	H1			H2			H3			H4		
	S1	S2	S3	S1	S2	S3	S1	S2	S3	S1	S2	S3
Ganoderic acid I	0.197±0.0153	0.297±0.0103	0.147±0.0053	0.297±0.0151	0.35±0.0016	0.143±0.0139	0.555±0.018	0.376±0.0045	0.301±0.0025	0.442±0.0027	0.303±0.0183	0.483±0.0608
lucidenic acid C	0.091±0.0038	0.084±0.0036	0.019±0.0031	0.002±0.0001	0.003±0.0008	0.01±0.0001	0.092±0.0066	0.033±0.0008	0.024±0.0002	0.14±0	0.101±0.0175	0.215±0.0106
ganoderic acid C2	0.357±0.0024	0.316±0.0056	0.081±0.0069	0.642±0.0008	0.73±0.0247	0.278±0.0769	0.485±0.0312	0.369±0.0086	0.502±0.0429	0.457±0.0002	0.242±0.0152	0.218±0.0089
lucidenic acid N	0.085±0.0022	0.093±0.0059	0.016±0.0014	0.003±0.0002	0.003±0.0005	0.024±0.0024	0.11±0.0075	0.062±0.0008	0.056±0.0013	0.113±0.0041	0.133±0.0235	0.271±0.0081
ganoderic acid C6	0.136±0.0018	0.077±0.0067	0.036±0.0054	0.42±0.01	0.25±0.0052	0.105±0.0017	0.033±0.0018	0.017±0.0013	0.053±0.0058	0.139±0.0082	0.053±0.0061	0.113±0.0182
ganoderic acid G	0.418±0.0009	0.419±0.0249	0.111±0.0032	0.759±0.0254	0.779±0.0142	0.236±0.0457	0.467±0.0154	0.298±0.0113	0.203±0.0085	0.668±0.0367	0.332±0.0095	0.237±0.0246
ganoderenic acid B	0.193±0.001	0.25±0.0017	0.068±0.0072	0.066±0.0013	0.029±0.008	0.012±0.0022	0.353±0.0154	0.317±0.0159	0.272±0.003	0.305±0.0096	0.219±0.0163	0.237±0.0198
ganoderic acid N	0.142±0.0069	0.216±0.0164	0.105±0.0014	0.273±0.0006	0.405±0.0096	0.132±0.0102	0.38±0.0242	0.179±0.0013	0.091±0.0021	0.37±0.0086	0.241±0.0094	0.296±0.0463
ganoderic acid B	0.454±0.0148	0.491±0.0168	0.089±0.0045	1.001±0.0788	1.099±0.0003	0.445±0.1168	0.553±0.0194	0.466±0.0031	0.499±0.0045	0.55±0.0399	0.299±0.0122	0.227±0.0154
ganoderenic acid H	0.023±0.0005	0.024±0.0006	0.016±0.0014	0.017±0.0007	0.011±0.0016	0.006±0.0007	0.031±0.0009	0.028±0	0.035±0.0012	0.035±0.0005	0.024±0.0034	0.035±0.0036
lucidenic acid E	0.141±0.0087	0.144±0.0073	0.025±0.0034	0±0	0±0	0.038±0.0118	0.051±0.0026	0.035±0.0001	0.039±0.0021	0.131±0.0001	0.147±0.0337	0.601±0.0104
ganoderic acid AM1	0.102±0.0011	0.117±0.0023	0.023±0.0016	0.146±0.0052	0.177±0.0129	0.062±0.0151	0.106±0.0062	0.105±0.0011	0.095±0.0035	0.102±0.0028	0.073±0.0027	0.041±0.0002
ganoderenic acid A	0.536±0.0217	0.699±0.0092	0.328±0.025	0.13±0.0011	0.208±0.0203	0.095±0.0241	1.301±0.0356	0.807±0.0393	0.469±0.0463	1.138±0.0219	0.741±0.0179	0.629±0.131
ganoderic acid K	0.077±0.0024	0.156±0.0025	0.075±0.004	0.096±0.0053	0.16±0.0049	0.129±0.0164	0.039±0.0018	0.097±0.0038	0.088±0.0004	0.057±0.0014	0.101±0.016	0.185±0.0021
lucidenic acid B	0.078±0.0004	0.11±0.0029	0.042±0.0087	0.002±0.0001	0.002±0.0005	0.005±0.0009	0.05±0.0026	0.011±0	0.008±0.0007	0.148±0.0038	0.073±0.0075	0.073±0.021
ganoderic acid A	1.861±0.2416	2.63±0.174	1.228±0.004	1.938±0.1306	2.99±0.1666	1.323±0.1942	3.006±0.0708	2.459±0.1676	1.328±0.027	3.962±0.1487	3.338±0.1143	1.664±0.2302
ganoderic acid H	0.401±0.0284	0.622±0.0171	0.182±0.0037	0.734±0.0805	1.006±0.0203	0.734±0.0638	0.259±0.0092	0.304±0.0082	0.317±0.0056	0.488±0.012	0.421±0.0546	0.721±0.0198
dehydro-lucidenic acid A	0±0	0±0	0±0	0.051±0.0019	0.052±0.0061	0.081±0.0131	0±0	0±0	0±0	0±0	0±0	0.002±0.0006
deacetyl-ganoderic acid F	0.052±0.0037	0.058±0.0018	0.033±0.0005	0.257±0.01	0.276±0.0203	0.113±0.0011	0.035±0.0031	0.009±0.0005	0.011±0.0007	0.263±0.0168	0.091±0.0018	0.07±0.0077
lucidenic acid A	0.334±0.0138	0.465±0.0123	0.228±0.0333	0±0	0±0	0±0	0.311±0.0286	0.092±0.0108	0.041±0.0019	0.743±0.0006	0.422±0.0111	0.715±0.1164
ganoderic acid D2	0.249±0.001	0.269±0.0223	0.128±0.0052	0.447±0.0178	0.613±0.0255	0.227±0.0069	0.233±0.0189	0.089±0.0022	0.053±0.0013	0.601±0.0574	0.25±0.0087	0.175±0.0159
ganoderenic acid D	0.492±0.0027	0.662±0.021	0.301±0.0002	0.087±0.0046	0.084±0.0103	0.006±0.0015	0.784±0.0282	0.506±0.0271	0.236±0.0229	0.925±0.0112	0.617±0.022	0.421±0.061
ganoderic acid C1	0.882±0.001	1.106±0.0628	0.649±0.0183	1.278±0.0504	2.11±0.0485	0.925±0.0674	1.166±0.0832	0.629±0.0003	0.422±0.0115	2.316±0.2908	1.669±0.208	0.9±0.098
lucidenic acid D	0.307±0.0154	0.406±0.0073	0.14±0.0024	0±0	0±0	0±0	0.07±0.0062	0.018±0.0023	0±0	0.435±0.0096	0.298±0.0284	0.559±0.1132
ganoderic acid F	0.54±0.01	0.808±0.0342	0.494±0.013	0.782±0.006	1.464±0.0301	0.776±0.0129	0.33±0.015	0.245±0.0038	0.085±0.004	1.098±0.0556	0.69±0.001	0.577±0.1026
ganoderic acid J	0.06±0.0039	0.123±0.004	0.072±0.0052	0.084±0.0016	0.126±0.0196	0.075±0.0014	0.155±0.0123	0.146±0.0022	0.095±0.0041	0.132±0.0079	0.111±0.0022	0.079±0.0124
danzhi acid E	0.019±0.0012	0.036±0.0004	0.023±0.0005	0.009±0.0002	0.012±0.0014	0.017±0.0008	0.017±0.0041	0.014±0.0011	0.013±0.0009	0.036±0.002	0.027±0.0042	0.032±0.0046
ganodermanontriol	0.09±0.0414	0.109±0	0.135±0.0072	0.077±0.0039	0.141±0.0156	0.105±0.0012	0.07±0.0062	0.046±0.0002	0.043±0.0012	0.014±0.0008	0.009±0.0004	0.014±0
ganoderic acid DM	0.083±0.0012	0.074±0.0018	0.056±0.0005	0.098±0.0003	0.103±0.0047	0.071±0.0016	0.373±0.0528	0.193±0.0283	0.148±0.012	0.049±0.0058	0.024±0.0004	0.036±0.0024

ganoderic acid TR	0.007±0.0002	0.013±0.0008	0.018±0.0012	0.016±0.0003	0.025±0.0024	0.022±0.0005	0.035±0.0035	0.034±0.003	0.042±0.0032	0.029±0.0021	0.02±0.0007	0.028±0.003
Tatol	8.405±0.3046	10.873±0.0497	4.867±0.0586	9.711±0.0328	13.208±0.2357	6.194±0.6709	11.45±0.5312	7.984±0.2418	5.569±0.08	15.887±0.1884	11.068±0.1019	9.855±0.4348
